# Iowa State Veterinary Medical Association

**Published:** 1898-03

**Authors:** James E. Brown


### IOWA STATE VETERINARY MEDICAL ASSOCIATION.

TENTH annual meeting called to order at the Savery House, Des Moines, January 12, 1898, at 10 A.M., by the President, G. A. Johnson. There were present: Drs. S. H. Johnson, W. B. Niles, A. T. Peters, F. M. Roys, P. Malcolm, C. E. Stewart, H. L. Stewart, J. G. Parslow, J. I. Gibson, J. W. Griffith, E. A. Buxton, J. A. Campbell, A. T. Peters, J. H. McLeod, P. O. Koto, W. F. Lagear, A. S. Brodie, G. A. Johnson, G. A. Scott, H. E. Talbot, J. L. Williamson, W. H. Austin, D. H. Miller, G. M. Walrod, C. J. Hinkley, R. A. Craig, J. E. Brown. Minutes of the previous meeting were read and approved. Dr. G. A. Scott was appointed to fill vacancy on Board of Censors.

President's address was delivered, and discussion followed.

Letters were read by the Secretary from members and invited guests expressing regret at their inability to be present, and wishing the Association a successful meeting. Also from Drs L. A. Thomas, E. Benor, W. L. Williams, and W. A. Heck.

Dr. Gibson moved that as Dr. Thomas had been at considerable expense as a member of the Legislative Committee and had been a creditable member of the Association, before taking up the M.D. profession, that his dues be credited up to him in full, and that he be made an honorary member; seconded and carried.

The resignation of Dr. W. A. Heck was accepted, and he was voted an honorary membership so long as he remained a non-resident of the State.

The Treasurer's report, showing a balance on hand (after all debts were paid) of $10.48 was read, and referred to an auditing committee. Drs. W. H. Austin, C. E. Stewart, and C. J. Hinkley were named as Auditing Committee.

Secretary's report was read, and discussions followed. Tuberculin and tuberculin-tests, mallein and mallein-tests were discussed; also the idea of making an effort to have veterinarians placed on the programme of Farmers' Institutes and stock meetings as a means of educating the farmers and stockmen along sanitary lines, and, also, of bringing the profession before the people in the light of being awake to their needs and working for the best interests of the live-stock industry and general public health.

Drs. G. P. Stalter, E. A. Buxton, A. L. Brodie, R. A. Craig, and F. M. Roys were elected to membership.

Dr. A. T. Peters, of Lincoln, Neb., was present and extended an invitation to our members to be present at the meeting of their State Association on January 18, 1898.

Dr. J. I. Gibson, State Veterinary Surgeon of Iowa, announced that the annual report of his office was ready for distribution and would be sent to anyone who desired a copy.

Meeting adjourned until 1.30 P.M.

*Afternoon Session.*

Meeting called to order by President Johnson.

Report of cases on "Diseases and New Treatment," was made by Dr.

W. B. Niles, chairman, and brought forth an animated discussion on the different subjects. Drs. Gibson and Austin reported an outbreak of an infectious catarrhal disease in cattle. Dr. Koto reported a case where a piece of glass, supposed to have entered the body by way of the stomach, was removed from a mare's tail. Dr. Peters reported a case where a whip-stalk had been used as a probang to unchoke a cow; a piece was broken off and passed into the stomach. Some two years later an abscess formed in the cow's flank, and on being opened the piece of whip was found and removed. Dr. Brown reported case in which a sharp-pointed, headless nail had been swallowed with food, by a cow, and passed from the stomach, through its wall, through the diaphragm, to the heart. Several other interesting cases were reported by Drs. Campbell, Walrod, Gibson, Miller, and others.

Report of Committee on Articles for Newspaper Publication was made by Dr. W. B. Niles, chairman.

Auditing Committee reported that the Treasurer's accounts had been examined and found correct. Report was adopted and the committee discharged.

Dr. Gibson moved that Dr. Niles be made a committee to assign papers and essayists for Farmers' Institutes, as suggested in Secretary's report; seconded and carried.

Dr. Gibson offered a resolution referring to the U. S. V. M. A. meeting at Omaha.

Dr. Brown moved that the chair appoint a committee of three on resolutions; seconded and carried. The Chair appointed Drs. W. B. Niles, S. H. Johnson, and J. H. McLeod.

A committee, consisting of Drs. Brown, Niles, and Gibson, was appointed to investigate and get prices on the printing of the reports of this meeting, and to report later.

Dr. S. H. Johnson moved that two delegates be elected by ballot to represent this Association at the U. S. V. M. A. meeting in Omaha; seconded and carried.

Meeting adjourned until 7.30 P.M.

*Evening Session.*

Joint meeting of I. S. V. M. A. and Iowa Agricultural Society was called to order by President Johnson.

Dr. J. I. Gibson was introduced and read a paper on "Actinomycosis." On finishing Dr. A. T. Peters was introduced, and read a paper on the subject "Immunity." After the reading of this paper a number of very fine stereopticon views, illustrating the two papers, were thrown on the canvas, and greatly enjoyed by all present. Discussion followed for a short time, and then President John Cownie, of the Agricultural Society, arose and extended a most cordial invitation to the I. S. V. M. A. members to attend their banquet. Dr. Gibson moved to accept the invitation; seconded and carried.

Adjournment was made until next morning at 9.30.

*Morning Session—Second Day.*

Meeting called to order by President Johnson at 9.30 o'clock.

Discussions were taken up on Drs. Gibson's and Peters' papers. They were principally along the lines of preparing serums, hog-cholera treatment, etc.

Dr. J. G. Parslow was introduced, and read a paper on the subject, "Reflex Paralysis, or Paralysis from Indigestion." Discussion followed.

Dr. J. W. Griffith then read a paper on "Dairy Sanitation." Discussion followed.

Dr. J. H. McLeod read a paper entitled, "Notes from Case-book." Discussion followed on the treatment of umbilical hernia.

Dr. Emmert, member of the Iowa State Board of Health and State Senator-elect, was present, and spoke on the prevalence of tuberculosis in the State, and of its spread through the importation of Eastern dairy-cattle into Iowa. The Doctor outlined a bill which he proposed to introduce into the State Legislature, that would require a certificate of tuberculin-test to accompany cattle shipped into the State. Discussions followed. Dr. Emmert also spoke of a bill intended to prevent the spread of hog-cholera, by strict quarantine measures, which he would probably introduce.

Adjournment was then made until 4 P.M., so that the members might attend the inaugural exercises of Governor-elect L. M. Shaw.

*Afternoon Session—4 o'clock.*

Meeting called to order by President Johnson.

Dr. S. H. Johnson then read a paper on the subject, "Removal of Clitoris to Overcome Viciousness." Discussion followed on the operation and results; also the difficult operations for ovariotomy. Discussion closed.

Election of officers was made the next order of business. This resulted in the election of: Dr. S. H. Johnson, Carroll, Iowa, President; Dr. G. A. Scott, Independence, Iowa, First Vice-President; Dr. J. G. Parslow, Marshalltown, Iowa, Second Vice-President; Dr. J. E. Brown, Oskaloosa, Iowa, Secretary and Treasurer; Dr. W. H. Austin, Newton, Iowa; Dr. D. H. Miller, Harlan, Iowa; Dr. J. H. McLeod, Charles City, Iowa, Board of Censors. Delegates to U. S. V. M. A. meeting: Dr. P. O. Koto, Forest City, Iowa; Dr. H. E. Talbot, Des Moines, Iowa.

The Committee on Printing of Reports of Meeting reported that the same might be printed in the State Agricultural Society's report, with a copy for each member, free of charge. The report was received and the Secretary instructed to prepare the report and papers for publication, and forward same to the Secretary of the Agricultural Society.

The Committee on Legislation reported, through its chairman, Dr. J. I. Gibson. Report was accepted and the committee discharged. Dr. Niles moved that the President and Secretary should constitute a committee to voice the sentiment of the Association, and coöperate with the State Veterinary Surgeon on legislative matters; seconded and carried.

The Committee on Resolutions reported the following:

WHEREAS, we learn from Dr. Peters that the meeting of the U. S. V. M. A. will be held in Omaha next September;

*Resolved*, (1) That we, the members of the I. S. V. M. A. rejoice with the Nebraska State Veterinary Medical Association and the veterinarians of the West in the location of the U. S. meeting, and pledge to the Nebraska Association our coöperative action in receiving the members of the U. S. Association. (2) That we recommend a joint meeting of this Association and the Nebraska Association on the first day of the U. S. meeting in Omaha.

WHEREAS, Senator Emmert, of the State Board of Health, has kindly appeared before the I. S. V. M. A. for the purpose of explaining proposed legislation, and asking for the coöperation of the Society; therefore, be it

*Resolved*, That the members of the Iowa State Veterinary Association, in convention assembled, express their great appreciation of the interest shown by Dr. Emmert in the live-stock industry of the State, and pledge him their hearty support and coöperation in securing the passage of the proposed bill to prevent the introduction of bovine tuberculosis into the State, and any other legislation along sanitary lines; be it

*Resolved*, That we extend our sincere thanks to Dr. Peters for the great interest he has taken in our meeting, for the reading of his very able paper on "Immunity," and for the exhibition of the specimen in connection therewith. Also to Dr. Scott for the use of the stereopticon; and be it further

*Resolved*, That the members of the I. S. V. M. A. extend their thanks to the State Agricultural Society for their kind invitation to their annual banquet, and for the general interest they have shown in this meeting; be it

*Resolved*, That this Association tender its thanks to the management of the Savoy Hotel for the courtesies extended to the Association and for the use of the room during the annual meeting.

W. B. NILES, J. H. McLEOD, S. H. JOHNSON,
Committee on Resolutions.

The resolutions were adopted and ordered spread upon the minutes.

Dr. J. I. Gibson read a paper; subject, "General Sanitation," and discussion followed.

Dr. Brown moved that the Committee on Disease and Treatment be continued another year; seconded and carried.

Dr. Gibson moved that the meeting adjourn, to meet in Des Moines next fall or winter, at the call of the President and Secretary; seconded and carried.

JAMES E. BROWN.

